# Genetic Effects and Heterosis of Yield and Yield Component Traits Based on *Gossypium Barbadense* Chromosome Segment Substitution Lines in Two *Gossypium Hirsutum* Backgrounds

**DOI:** 10.1371/journal.pone.0157978

**Published:** 2016-06-27

**Authors:** Botao Li, Yuzhen Shi, Juwu Gong, Junwen Li, Aiying Liu, Haihong Shang, Wankui Gong, Tingting Chen, Qun Ge, Chaoyang Jia, Yake Lei, Yushu Hu, Youlu Yuan

**Affiliations:** 1 Institute of Cotton Research, Chinese Academy of Agricultural Sciences/ State Key Laboratory of Cotton Biology, Key Laboratory of Biological and Genetic Breeding of Cotton, The Ministry of Agriculture, Anyang, Henan 455000, China; 2 Changde Agricultural Science Research Institute, Changde, Hunan 415000, China; 3 Zhoukou Academy of Agricultural Sciences, Zhoukou, Henan 466001, China; 4 Liaoning Provincial Institute of Cash Crops, Liaoyang, Liaoning 111000, China; National Key Laboratory of Crop Genetic Improvement, CHINA

## Abstract

We hybridized 10 chromosome segment substitution lines (CSSLs) each from two CSSL populations and produced 50 F_1_ hybrids according to North Carolina Design II. We analyzed the genetic effects and heterosis of yield and yield components in the F_1_ hybrids and parents in four environments via the additive-dominance genetic model. Yield and yield components of the CSSLs were controlled by combined additive and dominance effects, and lint percentage was mainly controlled by additive effects, but boll weight, boll number, seedcotton yield and lint yield were mainly controlled by dominance effects. We detected significant interaction effects between genetics and the environment for all yields traits. Similar interactions were detected between two CSSL populations (Pop CCRI 36 and Pop CCRI 45). Significant positive mid-parent heterosis was detected for all yield traits in both populations, and significant positive better-parent heterosis was also detected for all yield traits except lint percentage. The differences among parents were relatively small, but significant heterosis was detected for yield and yield components. Therefore, the relationship between heterosis and genetic distance for yield traits is complicated and requires further study. These CSSLs represent useful tools for improving yield and yield components in cotton.

## Introduction

Cotton (*Gossypium* spp.) is one of the most important cash crops and the leading fiber resource for the textile industry worldwide. Most global cotton production involves two cultivated species, *G*. *hirsutum* and *G*. *barbadense*. *G*. *hirsutum*, also known as Upland cotton, represents 95% of global cotton fiber production [1]. *G*. *barbadense*, another important tetraploid cultivated species, has excellent fiber quality and disease resistance compared to Upland cotton, which exhibits high yield but only moderate fiber quality [2, 3]. The narrow genetic base and limited genetic diversity of Upland cotton has become a serious concern for genetic improvement of lint yield and fiber quality in cotton breeding [4–6].

Interspecific introgression can be used to transfer valuable alien genes from *G*. *barbadense* germplasm for the improvement of Upland cotton species [3, 5, 7–10]. Chromosome segment substitution lines (CSSLs, often referred to as introgression lines), which include near-isogenic lines covering the entire genome of a crop, can be developed by crossing donor and recipient parents, backcrossing to the recipient parent and using molecular marker-assisted selection (MAS) [3, 11]. In this process, one or a few homozygous chromosome segments are derived from the donor parent and the rest of the genome is the same as that of the recipient parent. These lines serve as ideal tools for quantitative genetics, utilization of heterosis and gene pyramiding breeding [2, 3, 10, 12–14].

Cotton yield is still the primary objective of cotton breeding programs. The genetic improvement of cotton yield is usually difficult to achieve due to its complex relationship with yield components and genotype-by-environment interactions [15]. Many studies focusing on the genetic basis of yield and yield components in Upland cotton have produced different results depending on the materials and analysis methods utilized [15–23]. Luan and Guo [8] determined that boll weight is mainly controlled by additive effects, whereas boll number is controlled by dominance effects and lint percentage and yield are controlled by combined additive and dominance effects. Additionally, Shi et al. [9] found that significant additive and dominance effects control boll weight and lint percentage, while boll weight is mainly controlled by additive effects and lint percentage is controlled by combined additive and dominance effects in hybrid combinations between *G*. *hirsutum* and *G*. *barbadense*. Some evidence indicates that all yield traits of CSLs are controlled by combined additive and dominance effects [24]. Nevertheless, Zhang et al. [12] indicated that lint percentage is mainly controlled by additive effects and that boll number is mainly controlled by dominance effects. Such studies have mainly focused on population construction, phenotypic evaluation and QTL mapping of cotton CSSLs [2, 3, 10, 12–14], whereas the genetic effects of yield and yield components of the CSSLs were not analyzed in detail.

In this study, we developed two sets of CSSLs, Pop CCRI 36 (CCRI36 × Hai1 BC_5_F_3:5_) and Pop CCRI 45 (CCRI45 × Hai1 BC_4_F_3:5_). We randomly selected 10 CSSLs that each line has at least 3 chromosome segments, the segements of Hai1 are anchored. Each of these populations as parents for hybridization and produced 50 F1 hybrids according to North Carolina Design II. We measured and analyzed the genetic effects and heterosis of yield and yield component traits via the additive-dominance genetic model. The objectives of this study were to determine the genetic mechanism underlying yield and yield components of the CSSLs. The results of this study will facilitate the utilization of CSSLs in future cotton breeding programs.

## Materials and Methods

### Development of cotton CSSLs

**Fig 1:**
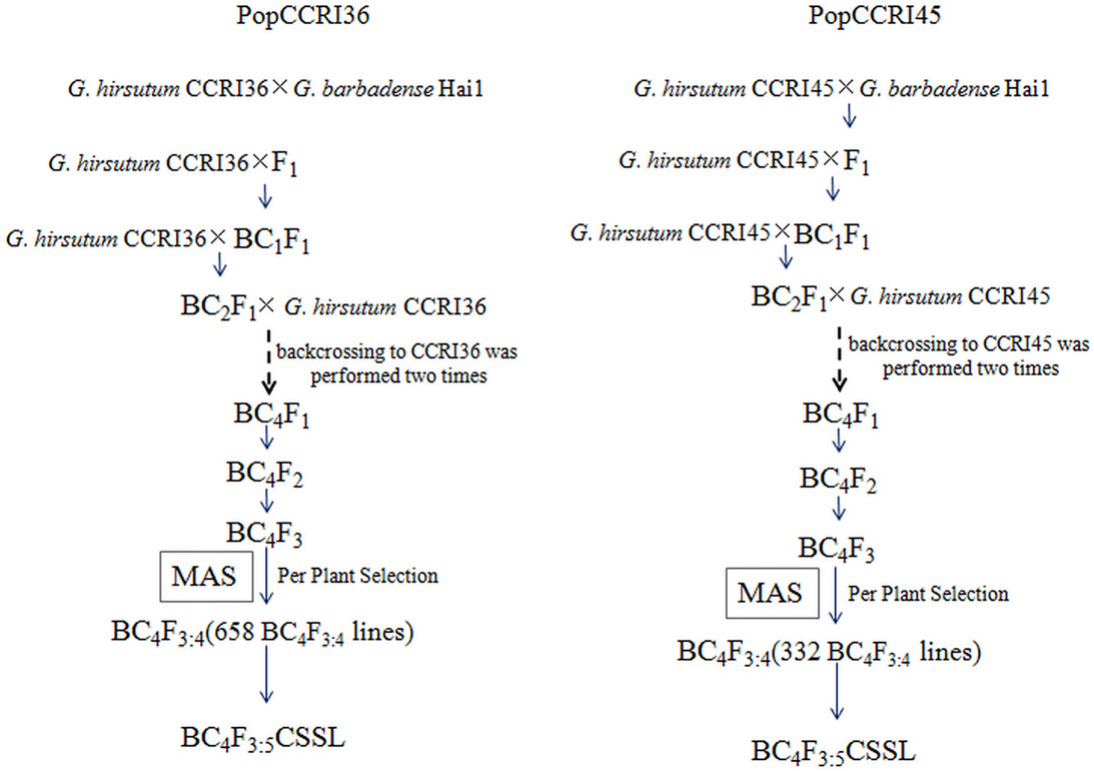
Breeding scheme for the development of the chromosome segment substitution lines (CSSLs).

PopCCRI36 (CCRI36 × Hai1 BC_5_F_3:5_): The F_1_ population was generated by a cross using early-maturing variety CCRI36 (Chinese Cotton Research Institute 36, *Gossypium hirsutum*) as the recipient and Hai1 (*Gossypium barbadense*) as the donor parent. The cross was performed in the summer of 2003 at the experimental station of the Institute of Cotton Research of CAAS, Anyang, Henan Province, China(36°05′5.78″N, 114°30′34.27″E). The development scheme for the CSSLs was as described [12]. The F_1_ (CCRI36 × Hai1) progeny were backcrossed to CCRI36 to produce BC_1_ in the winter of 2003 in Sanya, Hainan province(18°15′14.95″N, 109°30′28.78″E). The BC_1_ progeny were backcrossed to CCRI36 to produce BC_2_ in the summer of 2004 in Anyang (36°05′5.78″N, 114°30′34.27″E), and advanced backcross generations up to BC5 were similarly obtained. A total of 133 BC5 family lines were selfed and individually planted to produce BC_5_F_2_ in the summer of 2007 in Anyang (36°05′5.78″N, 114°30′34.27″E). BC_5_F_2_ progeny were selfed and individually planted to generate BC_5_F_3_ in the summer of 2008 in Anyang (36°05′5.78″N, 114°30′34.27″E). A total of 2,660 plants from 133 BC_5_F_3_ families were planted in the summer of 2009, and DNA samples were extracted from each plant to initiate MAS screening of *G*. *barbadense* introgressed chromosome segments. Phenotypic traits of 658 BC_5_F_3:4_ lines related to fiber yield and quality were evaluated in the summer of 2010 in Anyang (36°05′5.78″N, 114°30′34.27″E). Ten CCRI36 × Hai1 BC_5_F_3:5_ lines with good fiber quality and stable performance of yield traits were selected from PopCCRI36 and planted in the summer of 2011 in Anyang (36°05′5.78″N, 114°30′34.27″E) (Fig 1). Five lines, MBI9915, MBI9476, MBI9855, MBI9517 and MBI9913, were selected as the female parents and five lines, MBI9134, MBI9045, MBI9170, MBI9749 and MBI9755, were selected as the male parents. Twenty-five F_1_ hybrids were generated via North Carolina Design II in the summer of 2011 in Anyang (36°05′5.78″N, 114°30′34.27″E).

PopCCRI45 (CCRI45 × Hai1 BC_4_F_3:5_): The F_1_ population was generated by a cross using mid-maturing variety CCRI45 (Chinese Cotton Research Institute 45, *Gossypium hirsutum*) as the recipient and Hai1 as the donor parent in the summer of 2003 at the experimental station of the Institute of Cotton Research of CAAS, Anyang, Henan Province, China (36°05′5.78″N, 114°30′34.27″E). The development scheme for CSSLs was as described [13]. The F_1_ progeny of CCRI45 × Hai1 were backcrossed to CCRI45 to produce BC_1_ in the winter of 2003 in Sanya, Hainan province (18°15′14.95″N, 109°30′28.78″E). The BC_1_ progeny were backcrossed to CCRI45 to produce BC_2_ in the summer of 2004 in Anyang (36°05′5.78″N, 114°30′34.27″E), and advanced backcross generations up to BC_4_ were similarly obtained. A total of 116 BC_4_ family lines were selfed and individually planted to produce the BC_4_F_2_ population in the summer of 2007 in Anyang (36°05′5.78″N, 114°30′34.27″E). BC_4_F_2_ plants were selfed and individually planted to generate the BC_4_F_3_ population in the summer of 2008 in Anyang (36°05′5.78″N, 114°30′34.27″E). A total of 2,328 plants from 116 BC_4_F_3_ families were planted in the summer of 2009, and DNA samples were extracted from each plant to initiate MAS screening of *G*. *barbadense* introgressed chromosome segments. Phenotypic traits of 332 BC_4_F_3:4_ lines related to fiber yield and quality were evaluated in the summer of 2010 in Anyang (36°05′5.78″N, 114°30′34.27″E). Ten CCRI45 × Hai1 BC_4_F_3:5_ lines with good fiber quality and stable performance of yield traits were randomly selected and planted in the summer of 2011 in Anyang (36°05′5.78″N, 114°30′34.27″E) (Fig 1). Five lines, MBI7115, MBI7346, MBI7469, MBI7506and MBI7505, were selected as the female parents and five lines, MBI7049, MBI7541, MBI7455, MBI7251and MBI7311, were selected as the male parents. Twenty-five F_1_ hybrids were generated via North Carolina Design II in the summer of 2011 in Anyang (36°05′5.78″N, 114°30′34.27″E).

### Field experimental design and planting

The recipient parent (CCRI36), the donor parent (Hai1), 10 parents of CSSLs from PopCCRI36 and 25 F_1_ hybrids were grown in one location (Changde, Hunan Province, 29°02′17.64″N, 111°37′39.89″E) in 2012 and in three locations (Anyang, Henan Province, 36°05′5.78″N, 114°30′34.27″E; Changde, Hunan Province, 29°02′17.64″N, 111°37′39.89″E; Liaoyang, Liaoning Province, China, 41°15′50.63″N, 123°08′7.16″E) in 2013. All experiments were conducted in a randomized complete block design with two replications. Each plot contained two rows with the following layout: 5 m long and 0.8 m apart with 20 plants per row in Anyang, 36°05′5.78″N, 114°30′34.27″E; 5 m long and 1.0 m apart with 11 plants per row in Changde; 3 m long and 0.55 m apart with 15 plants per row in Liaoyang. Standard agronomic practices were employed during the growing season for all environments.

The recipient parent (CCRI45), the donor parent (Hai1), 10 parental lines of CSSLs from PopCCRI45 and 25 F_1_ hybrids were grown in two locations (Anyang, Henan Province; Changde, Hunan Province) in 2012 and in two locations (Anyang, Henan Province; Zhoukou, Henan Province, 33°38′40.39″N, 114°40′42.35″E) in 2013. All experiments were conducted in a randomized complete block design with two replications. Each plot contained two rows with the following layout: 5 m long and 0.8 m apart with 20 plants per row in Anyang, 36°05′5.78″N, 114°30′34.27″E; 5 m long and 1.0 m apart with 11 plants per row at Changde, 29°02′17.64″N, 111°37′39.89″E; 5 m long and 0.85 m apart with 20 plants per row in Zhoukou, 33°38′40.39″N, 114°40′42.35″E. Standard agronomic practices were employed during the growing season for all environments.

Thirty normal, open bolls from each plot were randomly sampled by hand, weighed and ginned on a laboratory ginning machine to determine boll weight (BW) and lint percentage (LP). The plots were harvested by hand, and the seedcotton was weighed and used to calculate seedcotton yields (SCY). Boll number (BN) per hectare was calculated by dividing SCY by boll weight [6, 15]. Lint yield (LY) per hectare was determined by multiplying SCY by lint percentage.

### Statistical methods and genetic models

The LSD (Least-Significant Difference) method was used for mean comparisons among F_1_ hybrids and parents (SAS 9.2, SAS Institute). An additive-dominance (AD) genetic model with genotype and environment interaction effects was employed for data analysis [25]. A mixed model, minimum norm quadratic unbiased estimation (MINQUE) approach was used to estimate genetic variance components. Adjusted unbiased prediction (AUP) was adopted to estimate genetic effects [26]. Jackknifing over blocks within environments was applied to calculate standard errors of variance components and genetic effects. An approximate t-test was used to test the significance of each parameter. Heterosis over mid-parent (MP) and better parent (HP) was calculated based on population mean [21]: Hpm (F_1_) = (F_1_-MP)/μ; Hpb (F_1_) = (F_1_-BP)/μ, where, F_1_ denotes F_1_ genotypic value; MP denotes the mid-parent value of both parents; BP is the genotypic value of the better parent; and μ denotes the average value of all parents and F_1_ combinations using North Carolina Design II. The length of substituted chromosome segments in CSSLs was estimated based on Graphical genotypes 32 [27].

## Results

### Distribution of Substitution Segments on Chromosomes in CSSLs

**Fig 2:**
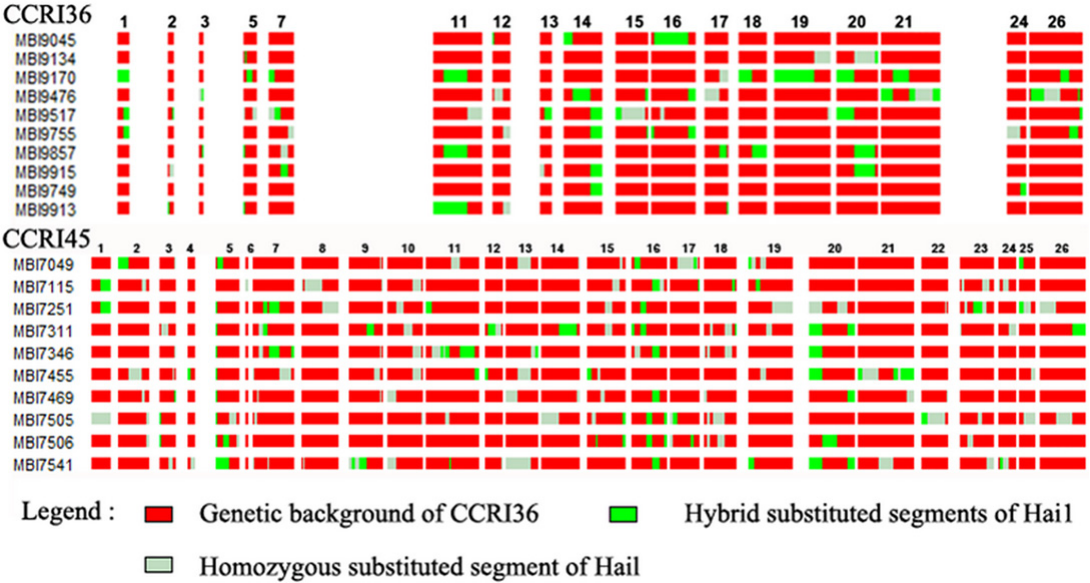
Graphical genotypes of the chromosome segment substitution lines (CSSLs).

Graphical genotypes of the CSSLs determined using 459 SSR markers distributed across 26 chromosomes are shown in Fig 2. Each line contained a substituted segment of a particular chromosomal region and/or additional small segments in non-target regions. There were 113 donor segments in total with an average of 11.3 introgressions per CSSL in CRRI36 and there were 240 donor segments in total with an average of 24 introgressions per CSSL in CRRI45. The quality of CSSLs could be ascertained from the number and amount of introgressed donor segments(Fig 2 and S1 Excel).

### Mean value comparisons of parents and F_1_ hybrids

Mean value comparisons of recipient (CCRI 36 and CCRI 45), donor parents, CSSLs parents and F_1_ hybrids are shown in Table 1 and S2 Excel. Compared to CCRI36, Hai1 produced significantly lower boll weight (BW), lower lint percentage (LP), lower boll number (BN) and lower seedcotton yield (SCY) and lint yield (LY). All yield traits of parents selected from CSSLs were lower on average than those of CCRI36, but the differences were not significant for any yield trait except LP. Compared to CCRI36, F_1_ had higher values for all yield traits except LP, and the difference in SCY was significant. In the F_1_ population from the converse cross, the parents had lower values, on average, for all yield traits; the difference was significant for SCY and LY. These results indicate that heterosis was detected for all yield traits, especially SCY and LY.

**Table 1:** Phenotypic means of yield and yield components for parents and F_1_.

Population	Materials	BW (g)	LP (%)	BN (10^5^/hm^2^)	SCY (kg/hm^2^)	LY (kg/hm^2^)
PopCCRI36	MBI9915	4.51	34.56	4.94	2242	781
	MBI9476	4.76	31.15	3.83	1820	564
	MBI9855	4.87	35.08	4.81	2313	816
	MBI9517	5.03	35.39	4.99	2491	879
	MBI9913	5.37	35.56	4.12	2185	776
	MBI9134	4.41	30.71	4.92	2131	653
	MBI9045	4.87	34.82	4.50	2170	756
	MBI9170	4.74	33.15	3.81	1772	581
	MBI9749	5.04	34.27	5.55	2771	953
	MBI9755	4.69	33.32	4.28	1992	666
	CCRI36	5.01	36.65	4.52	2248	824
	Hai1	3.18	34.59	2.10	667	228
	Mean of CSSLs	4.83	33.80	4.58	2189	743
	Mean of F_1_	5.22	35.01	5.47	2850	1001
	LSD (0.05)	0.45	1.65	1.15	597	223
PopCCRI45	MBI7115	5.20	35.18	4.10	2143	762
	MBI7346	5.59	31.40	4.86	2710	855
	MBI7469	5.03	32.49	4.59	2311	754
	MBI7506	5.58	31.61	4.27	2383	756
	MBI7505	5.84	32.10	4.12	2439	792
	MBI7049	5.14	36.59	4.72	2456	908
	MBI7541	5.03	34.55	4.75	2427	844
	MBI7455	5.36	36.22	3.68	1992	723
	MBI7251	4.89	29.57	3.06	1497	446
	MBI7311	4.96	33.21	4.80	2376	792
	CCRI45	5.59	33.69	4.77	2670	901
	Hai1	3.06	33.67	2.98	897	302
	Mean of CSSLs	5.26	33.29	4.30	2273	763
	Mean of F_1_	5.79	33.90	4.93	2859	970
	LSD (0.05)	0.38	1.80	0.69	460	169

Note: PopCCRI36, CCRI36 × Hai1 BC_5_F_3:5_; PopCCRI45, CCRI45 × Hai1 BC_4_F_3:5_; BW, boll weight; LP, lint percentage; BN, boll number; SCY, seedcotton yield; LY, lint yield; LSD (0.05) was used to compare mean values

Compared to CCRI45, Hai1 had lower values for all yield traits, and significant differences were detected for all traits except LP. The values of parents selected from the CSSLs were lower on average than those of CCRI45 for all yield traits, but the differences were not significant. Compared to CCRI45, F_1_ had higher values for all yield traits, but the differences were not significant. Compared to F_1_, the values of the parents were lower on average for all yield traits, with significant differences for BW, SCY and LY. These results indicate that heterosis was detected for all yield traits, especially BW, SCY and LY.

The results indicate that significant differences existed for all yield traits between the recipient parent (CCRI36 or CCRI45) and donor parent (Hai1), but the differences were relatively small on average between the recipient parent and CSSLs. In addition, the F_1_ hybrids exhibited heterosis for all yield traits for both populations (PopCCRI36and PopCCRI45), especially SCY and LY.

### Variance components

We estimated variance components for yield and yield components; the estimated ratios of variance components to phenotypic variance are shown in Table 2. In Pop CCRI36, significant additive effects were detected for all yield traits except SCY. Dominance effects were significant for all yield traits. Additive-by-environment interaction effects were significant for LP and LY. Dominance and environment interaction effects were significant for all yield traits except LP. Dominance effects played a major role in genetic variation for BW, BN, SCY and LY. Additive genetic variance contributed 48.3% of phenotypic variance for LP. In addition, the results indicate that genotypic variances (A and D) were more important than genetic-by-environment variances (AE and DE) for all yield traits.

**Table 2:** Estimated ratios of variance components to phenotypic variance.

Population	Parameter	BW (g)	LP (%)	BN (10^5^/hm^2^)	SCY (kg/hm^2^)	LY (kg/hm^2^)
PopCCRI36	V_A_/Vp	0.068*	0.483**	0.092*	0.040	0.093*
	V_D_/Vp	0.492**	0.205**	0.255**	0.428**	0.449**
	V_AE_/Vp	0.019	0.087**	0.066	0.055	0.062*
	V_DE_/Vp	0.147**	0.052	0.193*	0.166*	0.128*
	V_e_/Vp	0.271**	0.17**	0.394**	0.308**	0.265**
PopCCRI45	V_A_/Vp	0.093*	0.520**	0.126*	0.051	0.064
	V_D_/Vp	0.479**	0.041	0.234**	0.387**	0.341**
	V_AE_/Vp	0.053*	0.055*	0.127**	0.124**	0.150**
	V_DE_/Vp	0.115*	0.169**	0.180**	0.156**	0.173**
	V_e_/Vp	0.258**	0.212**	0.333**	0.280**	0.270**

Note: PopCCRI36, CCRI36 × Hai1 BC_5_F_3:5_; PopCCRI45, CCRI45 × Hai1 BC_4_F_3:5_; V_A_, additive genetic variance; V_D_, dominance genetic variance; V_P_, phenotypic variance; V_AE_, additive by environment variance; V_DE_, dominance by environment variance; V_e_, residual variance. BW, boll weight; LP, lint percentage; BN, boll number; SCY, seedcotton yield; LY, lint yield;

**, *: significant at the 0.01 and 0.05 level, respectively

In PopCCRI45, additive effects were significant for all yield traits except SCY and LY. Significant dominance effects were detected for all yield traits except LP. Additive-by-environment and dominance-by-environment interaction effects were significant for all yield traits. Dominance effects played a major role in genetic variation for BW, BN, SCY and LY. Additive genetic variance contributed 52.0% of phenotypic variance for LP. In addition, the results indicate that genotypic variances (A and D) were more important than genetic-by-environment variances (AE and DE) for all yield traits.

In general, the yield traits of CSSLs from both PopCCRI36 and PopCCRI45 were controlled by both additive and dominance effects, and significant genetic-by-environment interaction effects were detected. In addition, LP was mainly controlled by additive effects, but BW, BN, SCY and LY were mainly controlled by dominance effects. Therefore, selection for LP should be effective in early generations. We detected a similar genetic mechanism for all yield traits of CSSLs in two populations (PopCCRI36 and PopCCRI45) with different genetic backgrounds, although the proportions of genetic variance components were slightly different.

### Additive effects

Determining additive effects is important for identifying desirable general combiners to improve traits of interest (Wu et al. 2010). The predicted additive effects for yield and yield components are shown in Table 3. In PopCCRI36, the number of parents with significant positive additive effects were as follows: three for BW (MBI9517, MBI9913 and MBI9749), six for LP (MBI9517, MBI9915, MBI9855, MBI9913, MBI9045 and MBI9749) and three for BN (MBI9517, MBI9045 and MBI9749). These results indicate that these lines can be used as general combiners to improve BW, LP and BN, respectively. In addition, two lines, MBI9517 and MBI9749, had significant positive additive effects for all yield components, indicating that both lines can be used as combiners to simultaneously improve all yield components, ultimately improving seedcotton yield and lint yield.

**Table 3:** Predicted values of additive effects for parents.

Population	Materials	BW (g)	LP (%)	BN (10^5^/hm^2^)	SCY (kg/hm^2^)	LY (kg/hm^2^)
PopCCRI36	MBI9915	-0.074*	0.657**	0.074	-3.404	11.154
	MBI9476	0.036	-1.343**	-0.219**	-56.401	-49.167**
	MBI9855	-0.015	0.421**	0.047	7.615	9.721
	MBI9517	0.042*	0.504**	0.172*	78.520	43.58**
	MBI9913	0.108*	0.889**	-0.133*	-3.580	13.774
	MBI9134	-0.09	-1.913**	0.005	-39.234	-52.172*
	MBI9045	0.004	0.685**	0.051**	20.417*	22.085**
	MBI9170	-0.018*	0.158	-0.254**	-98.332*	-43.456**
	MBI9749	0.050*	0.440**	0.392**	161.586	84.541**
	MBI9755	-0.044*	-0.500*	-0.135*	-67.198	-40.066**
PopCCRI45	MBI7115	0.014	0.710**	-0.126**	-45.726	-6.636
	MBI7346	0.073*	-0.838**	0.336**	155.44*	42.754**
	MBI7469	-0.087*	0.190	0.063*	-15.057	-2.848
	MBI7506	0.100*	-0.823**	-0.048*	15.295*	-6.597
	MBI7505	0.155**	-0.819**	-0.072	30.963	-0.929
	MBI7049	-0.066*	1.316**	0.157	38.609	34.685
	MBI7541	-0.110*	0.541*	0.112**	5.849	10.288
	MBI7455	0.063	1.444**	-0.143*	-27.791	9.573
	MBI7251	-0.092	-1.692**	-0.384**	-176.429*	-85.121*
	MBI7311	-0.051*	-0.028	0.106*	18.832	4.827

Note: PopCCRI36, CCRI36 × Hai1 BC_5_F_3:5_; PopCCRI45, CCRI45 × Hai1 BC_4_F_3:5_; BW, boll weight; LP, lint percentage; BN, boll number; SCY, seedcotton yield; LY, lint yield;

**,*: significant at the 0.01 and 0.05 level, respectively

In PopCCRI45, the number of parents with significant positive additive effects were as follows: three for BW (MBI7346, MBI7505 and MBI7506), four for LP (MBI7115, MBI7049, MBI7541 and MBI7455) and four for BN (MBI7346, MBI7469, MBI7541 and MBI7311). These results indicate that these lines can be used as general combiners to improve BW, LP and BN, respectively. In addition, MBI7346 had significant positive additive effects for BW and BN, indicating that this line could be used as a combiner to improve seedcotton yield and lint yield.

### Dominance effects

In PopCCRI36, 10 and 11 hybrids had significant positive heterozygous dominance effects for SCY and LY, respectively (Table 4). In addition, the hybrid MBI9915 × MBI9749 had the highest value of predicted dominance effect for SCY and LY, i.e., 440.4 and 211.6 kg/hm^2^, respectively. In PopCCRI45, significant positive heterozygous dominance effects for SCY and LY were detected in 10 and 10 hybrids, respectively (Table 5). The hybrid MBI7346 × MBI7541 had the highest value of dominance effect for SCY, i.e., 457.1 kg/hm^2^, and the hybrid MBI7346 × MBI7455 had the highest value of dominance effect for LY, i.e., 176.1 kg/hm^2^. These results indicate that these hybrids, which had significant positive heterozygous dominance effects for SCY and LY, could be used to increase cotton yield during production.

**Table 4:** Heterozygous dominance effects of F_1_ for yield and yield components in PopCCRI36.

Hybrid	BW (g)	LP (%)	BN (10^5^/hm^2^)	SCY (kg/hm^2^)	LY (kg/hm^2^)
MBI9915×MBI9134	0.311**	-0.669	-0.315	-27.5	-33.3
MBI9915×MBI9045	-0.028	0.666	0.333*	158.3	74.9
MBI9915×MBI9170	0.042	0.823*	0.183	109.3	53.2
MBI9915×MBI9749	0.221**	1.457**	0.595**	440.4**	211.6**
MBI9915×MBI9755	0.127*	1.306*	0.226	178.7	96.1
MBI9476×MBI9134	0.085	-0.709*	0.440*	283.6*	71.5
MBI9476×MBI9045	0.392**	0.174	0.298	357.6*	124.6*
MBI9476×MBI9170	0.231**	1.505**	0.125	161.2	87.9*
MBI9476×MBI9749	0.271**	0.480*	0.244	271.0*	96.6*
MBI9476×MBI9755	0.097	-0.916**	0.213	149.6	26.0
MBI9855×MBI9134	0.180	-0.230	0.482	368.0*	115.5*
MBI9855×MBI9045	0.058	0.447*	0.338	215.6	89.4*
MBI9855×MBI9170	-0.018	0.618*	-0.058	-46.0	-4.2
MBI9855×MBI9749	0.075	0.642*	0.550*	376.3*	160.1**
MBI9855×MBI9755	0.102*	-0.202	-0.215	-94.4	-44.9
MBI9517×MBI9134	-0.052	0.503*	0.118	49.32	29.1
MBI9517×MBI9045	0.265**	0.846	0.470	407.0*	177.9*
MBI9517×MBI9170	0.277*	0.604	0.303	293.3*	123.9*
MBI9517×MBI9749	-0.011	-0.512*	0.154	78.7	7.1
MBI9517×MBI9755	0.070	-0.384	0.511**	329.3**	102.7**
MBI9913×MBI9134	0.226*	-0.339	-0.116	60.7	9.4
MBI9913×MBI9045	-0.116	1.053**	0.292	115.4	74.4
MBI9913×MBI9170	0.113	0.460	0.574*	384.1**	153.2**
MBI9913×MBI9749	0.030	1.017**	0.316	233.3	122.4
MBI9913×MBI9755	0.121	0.465	0.230	184.5	75.7

Note: BW, boll weight; LP, lint percentage; BN, boll number; SCY, seedcotton yield; LY, lint yield.

**,*: significant at the 0.01 and 0.05 level, respectively

**Table 5:** Heterozygous dominance effects of F_1_ for yield and yield components in PopCCRI45.

Hybrid	BW (g)	LP (%)	BN (10^5^/hm^2^)	SCY (kg/hm^2^)	LY (kg/hm^2^)
MBI7115×MBI7049	0.155	0.292	0.387	307.0*	118.4*
MBI7115×MBI7541	0.129	-0.444*	0.052	69.7	5.1
MBI7115×MBI7455	0.286*	0.372	0.165	213.3*	94.0**
MBI7115×MBI7251	0.180*	0.349	0.357*	274.0**	94.4*
MBI7115×MBI7311	0.320**	-0.296	-0.442*	-124.0	-56.9
MBI7346×MBI7049	-0.162*	-0.218	0.598**	267.3*	77.0*
MBI7346×MBI7541	-0.018	-0.078	0.781*	457.1*	142.3*
MBI7346×MBI7455	0.260*	0.586	0.455*	420.8**	176.1**
MBI7346×MBI7251	0.299*	0.239	0.086	159.2	53.3
MBI7346×MBI7311	0.321**	-0.029	-0.094	108.3	28.6
MBI7469×MBI7049	0.268*	0.258	0.054	157.7*	68.3**
MBI7469×MBI7541	0.130	0.503**	-0.296	-124.9	-23.3
MBI7469×MBI7455	-0.067	0.733*	0.111	21.3	36.5
MBI7469×MBI7251	0.398**	-0.06	0.340	359.3**	101.2*
MBI7469×MBI7311	-0.077	0.85	0.494*	255.0	122.7*
MBI7506×MBI7049	0.121	0.379*	-0.173	-57.7	-6.9
MBI7506×MBI7541	0.117**	0.281	0.258	225.7	91.4
MBI7506×MBI7455	0.331**	-0.295	0.223	274.7*	80.8
MBI7506×MBI7251	-0.001	-0.214	0.148	61.9	4.6
MBI7506×MBI7311	0.365**	0.117	0.188	294.3	98.3
MBI7505×MBI7049	0.200*	-0.439	0.147	187.3	41.0
MBI7505×MBI7541	0.107	0.329	-0.115	-10.6	11.6
MBI7505×MBI7455	0.300*	-0.217	0.295	299.2	86.3
MBI7505×MBI7251	0.045	-0.027	0.067	37.6	4.9
MBI7505×MBI7311	0.140	-0.030	0.401*	313.2**	97.1*

Note: BW, boll weight; LP, lint percentage; BN, boll number; SCY, seedcotton yield; LY, lint yield.

**,*: significant at the 0.01 and 0.05 level, respectively

In summary, positive heterozygous dominance effects were widely detected for all yield traits among the crosses, suggesting that it might be possible to utilize the heterosis for certain traits in some of these hybrids.

### Analysis of heterosis

As shown in Table 6, significant positive middle-parent (MP) heterosis was detected for all yield traits in both PopCCRI36 and PopCCRI45. The highest MP heterosis in PopCCRI36 was recorded for LY (30.2%), followed by SCY, BN, BW and LP (26.5%, 16.9%, 8.4% and 3.6%, respectively). The highest MP heterosis in PopCCRI45 was recorded for LY (23.7%), followed by SCY, BN, BW and LP (23.1%, 13.2%, 10.3% and 1.2%, respectively). The progeny from most of the 25 crosses from both PopCCRI36 and PopCCRI45 exhibited significant positive MP heterosis for these five traits (Table 6). In terms of heterosis over best parent (BP), significant positive BP heterosis was observed for BW, BN, SCY and LY, while LP had slight positive BP heterosis in PopCCRI36 and significant negative BP heterosis in PopCCRI45. The highest BP heterosis in PopCCRI36 was recorded for lint yield (24.2%), followed by SCY, BN, BW and LP (22.6%, 12.0%, 5.9% and 0.6%), respectively. The highest BP heterosis in PopCCRI45 was recorded for SCY (18.7%), followed by LY, BW, BN and LP (18.6%, 7.3%, 7.3% and -3.0%, respectively). The progeny from most of the 25 crosses from both of PopCCRI36 and PopCCRI45 had significant positive BP heterosis for all yield traits except LP.

**Table 6:** Mid-parent population mean heterosis and surpassing parental heterosis of yield and yield components.

Population	Trait	Hpm(F_1_)				Hpb(F_1_)			
		Mean	Range	+N	-N	Mean	Range	+N	-N
PopCCRI36	**BW**	0.084**	0.013~0.154	25(24)	0(0)	0.059**	-0.026~0.138	24(19)	1(1)
	**LP**	0.036**	-0.006~0.086	22(20)	3(0)	0.006*	-0.067~0.077	13(10)	12(9)
	**BN**	0.169**	0.012~0.252	25(23)	0(0)	0.120*	0.009~0.079	25(13)	0(0)
	**SCY**	0.265**	0.124~0.374	25(25)	0(0)	0.226**	0.087~0.333	25(16)	0(0)
	**LY**	0.302**	0.119~0.455	25(24)	0(0)	0.242**	0.067~0.334	25(18)	0(0)
PopCCRI45	**BW**	0.103**	0.027~0.155	25(25)	0(0)	0.073**	-0.003~0.141	24(21)	1(0)
	**LP**	0.012**	-0.016~0.046	19(16)	6(2)	-0.030**	-0.086~0.041	2(2)	23(21)
	**BN**	0.132**	-0.036~0.266	23(23)	2(1)	0.073**	-0.099~0.263	22(16)	3(2)
	**SCY**	0.231**	0.075~0.378	25(25)	0(0)	0.187**	0.058~0.319	25(24)	0(0)
	**LY**	0.237**	0.088~0.428	25(25)	0(0)	0.186**	0.046~0.376	25(23)	0(0)

Note: PopCCRI36, CCRI36 × Hai1 BC_5_F_3:5_; PopCCRI45, CCRI45 × Hai1 BC_4_F_3:5_; Hpm(F_1_): Average mid-parent population mean heterosis in F_1_; Hpb(F_1_): surpassing parental heterosis in F_1_;+N: number of combinations with positive heterosis; -N: number of combinations with negative heterosis; (): number of combinations attaining significantly positive or negative heterosis. BW, boll weight; LP, lint percentage; BN, boll number; SCY, seedcotton yield; LY, lint yield;

**,*: significant at the 0.01 and 0.05 level, respectively

These results indicate that significant positive MP and BP heterosis were detected for BW, BN, SCY and LY in both PopCCRI36 and PopCCRI45, and LP had significant positive MP heterosis. These results are in good agreement with the observed positive heterozygous dominance effects on these traits.

## Discussion

### Genetic effects of yield-related traits

Boll weight, lint percentage and boll number are three important components of lint yield in Upland cotton (*Gossypium hirsutum* L.). Selecting high-yielding lines or hybrids depends on the ability to dissect the genetic effects of yield and its component traits [15]. Many genetic studies have focused on yield and yield components in cotton, which produced diverse results due to the different materials and analysis methods employed [15–23]. In this study, we found that all yield traits of the CSSLs examined were controlled by combined additive and dominance effects, whereas LP was primarily controlled by additive effects and BW, BN, SCY and LY were mainly controlled by dominance effects. In addition, we detected significant interaction effects between genetics and the environment in the CSSLs for all yields traits. We detected a similar genetic mechanism for yield traits in two CSSL populations with different genetic backgrounds (PopCCRI36 and PopCCRI45), although the genetic variance component ratios were slightly different These results are mostly consistent with the results of Jenkins et al. [24], Li et al. [20] and Liu et al. [22], although the results disagree with those of Saha et al. [5], Luan and Guo [8] and Jenkins et al. [24]. It is generally accepted that yield traits are mainly controlled by non-additive effects, whereas LP is controlled by additive effects [16]. In addition, it is efficient to select for LP in early generations in cotton breeding programs, whereas other yield traits should be selected for in later generations. The interaction effects of genetics and the environment should also be considered.

### Heterosis of yield traits and genetic difference

The relationship between parental genetic distance and heterosis in cotton has been intensively investigated. Within a restricted range, greater genetic distance is associated with greater heterosis but over this range, heterosis decreases while genetic distance increases [28]. Yang et al. [29] reported that heterosis of yield and yield components is significantly correlated with genetic distance. In this study, we used CSSLs as parents to produce F_1_ hybrids. The genetic difference between parent lines and CSSLs was relatively small, as these lines were in the same genetic background, Upland cotton (*G*. *hirsutum* L.), with a few chromosome substitution segments from *G*. *barbadense* present in each CSSL (data not shown). Significant positive MP heterosis was detected for all yield traits in both PopCCRI36 and PopCCRI45, and significant positive HP heterosis was also detected for all yield traits except LP. These results are in good agreement with those of Li et al. [20], Liu et al. [22] and Luan and Guo [8]. The obvious heterosis of yield traits in the CSSLs suggests that these lines can be utilized for genetic improvement of yield in cotton.

### Potential of CSSLs in cotton breeding

Genetic diversity is considered to be a critical issue in cotton breeding programs [1, 15]. Introgressing useful alleles from *G*. *barbadense* into a *G*. *hirsutum* background is a useful approach for developing CSSLs [2, 3, 10]. In this study, we used CSSLs in which one or a few homozygous chromosome segments were derived from the donor parent but the rest of the genome was the same as that of the recipient parent. Based on the genetic mechanism uncovered in this study, it would be possible to breed cotton cultivars with high yield and superior fiber quality using CSSLs. Our results indicate that some CSSLs exhibited desirable additive effects for all yield traits and could therefore be used as parents to improve yield and yield components. Our results also show that positive heterozygous dominance effects and heterosis of yield and yield components widely existed in both PopCCRI36 and PopCCRI45, suggesting that hybrid production is another useful approach to cotton yield improvement.

## Conclusions

Yield and yield component of the CSSLs were controlled by combined additive and dominance effects, and LP was mainly controlled by additive effects, but BW, BN, SCY and LY were mainly controlled by dominance effects. Meanwhile, significant interaction effects between genetics and the environment were detected for all yields traits in the CSSLs. A similar genetic mechanism contributed to yield traits in two CSSL populations (PopCCRI36 and PopCCRI45) with different genetic backgrounds. Significant positive MP heterosis was detected for all yield traits in both PopCCRI36 and PopCCRI45, and significant positive HP heterosis was also detected for all yield traits except LP. The difference among parent lines selected from the CSSLs was relatively small, but widespread, significant heterosis was detected for yield and yield components. Therefore, CSSLs are useful genetic materials for improving yield and yield components in cotton breeding programs.

## Supporting Information

S1 Excel Distribution of Substitution segments (homozygous and heterozygous) in each CSSL for all 26 chromosomes. (XLS)

S2 Excel Different CSSLs for five agronomic traits of date. (XLSX)
